# A Case of Unilateral Brachial Plexus Injury Caused by First-Rib Stress Fractures Presenting With an Uncontrollable Involuntary Movement of the Neck

**DOI:** 10.7759/cureus.66649

**Published:** 2024-08-11

**Authors:** Kazuki Yokokawa, Minoru Yamada, Syuuichirou Suzuki, Shin Hisahara

**Affiliations:** 1 Department of Neurology, School of Medicine, Sapporo Medical University, Sapporo, JPN

**Keywords:** first-rib, nerve conduction study, involuntary movement, stress fracture, brachial plexopathy

## Abstract

Stress fracture of the first rib is a rare but an important cause of brachial plexopathy. Here, we describe a patient with a unilateral brachial plexus injury presenting with involuntary neck movements. A 22-year-old man with cervical involuntary movements for 10 months was diagnosed with tardive dyskinesia. After admission, he abruptly noticed that he could not lift his right arm. The electrophysiological study revealed weakness of the right deltoid and brachioradialis, but normal findings in the other muscles innervated by the right C5 segment. Chest computed tomography showed fractures of the first rib on both sides, with callus formation. Based on the involvement of muscles innervated by the right axillary and radial nerves and presence of callus at the first rib, a diagnosis of right posterior cord entrapment was made. In the present case, intermittent strong contraction of muscles due to dyskinesia may have caused the stress fractures.

## Introduction

Stress fracture of the first rib is a rare but an important cause of brachial nerve injury. Most of the cases reported are related to sports that require a heavy use of the upper extremities [1,2]. The posterior cord of the brachial plexus lies posterior to the second part of the axillary artery [3]. The posterior cord is formed by the union of the posterior division of the upper, middle, and lower trunk of the brachial plexus. The posterior cord of the brachial plexus after giving upper subscapular, thoracodorsal, lower subscapular, and axillary nerve in the axilla continues distally as the radial nerve. Here, we report a case of stress fractures of the first rib with unilateral posterior cord paralysis caused by an uncontrollable involuntary movement of the neck.

## Case presentation

A 22-year-old right-handed man was admitted to the psychiatric department of our hospital for generalized involuntary movements for the past 10 months. He had no developmental problems, but had been a restless child from early childhood with trouble keeping up with schoolwork. He stopped going to school at the age of 17 because of relationship troubles. He had no history of pathologic fractures, and had no particular sport history. He was diagnosed with depression at the age of 20 and was prescribed multiple psychotropic medications. Despite the attempt to find out the detailed drug history, access was not possible. He exhibited involuntary movements characterized by irregular flexion and extension of the neck and trunk, with these involuntary movements disappearing during sleep. He had clear consciousness during the involuntary movements. The involuntary movements frequently interfered with eating, and during severe episodes, he sometimes struck his head against walls or other objects. His involuntary movements were diagnosed as tardive dyskinesia, after excluding various primary involuntary movement disorders. Drug therapies including tetrabenazine were tried and were partially successful, but symptoms persisted.

**Figure 1:**
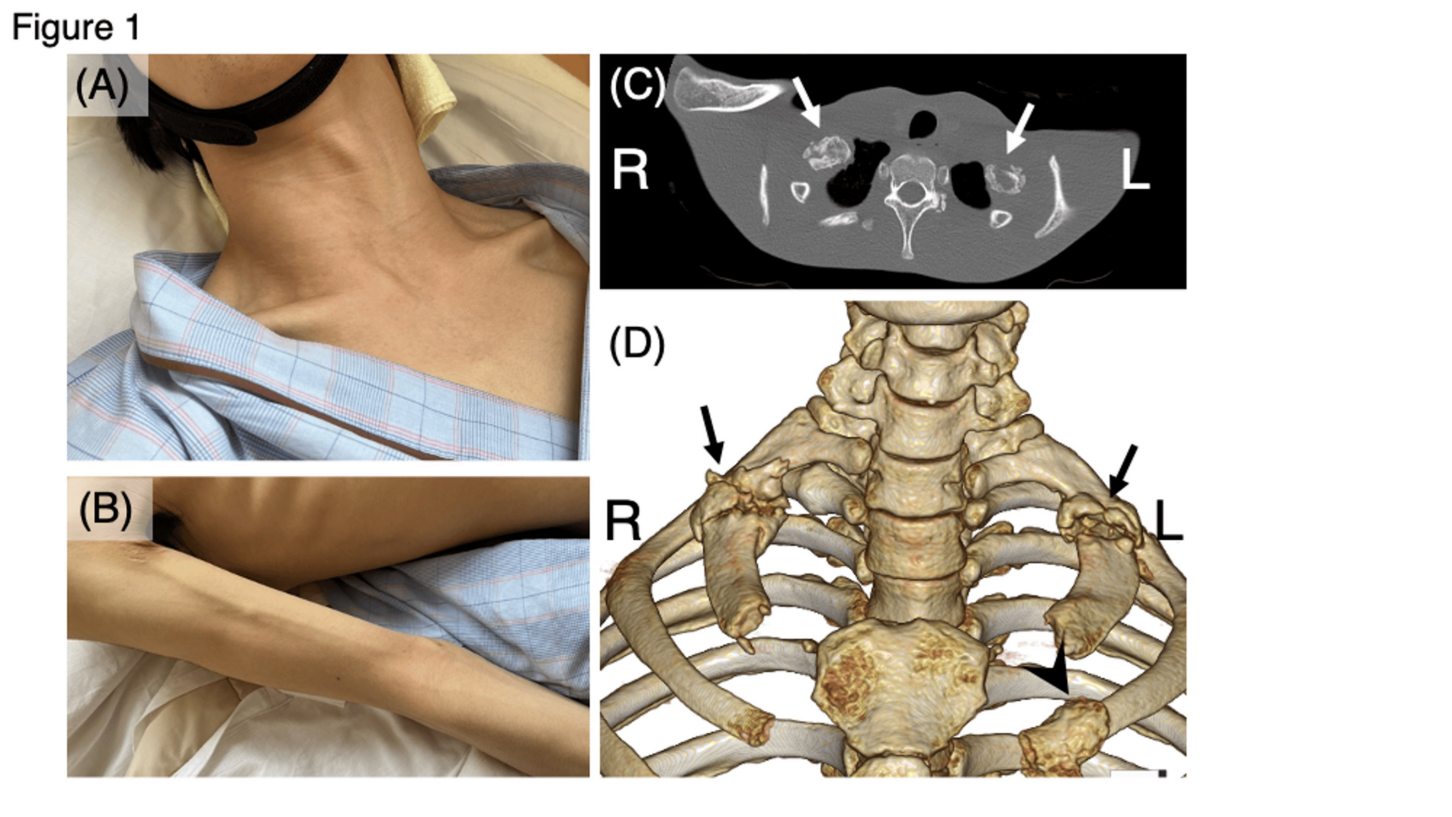
Clinical photographs and computed tomography findings (from the neck to the chest) Clinical photographs of the patient's neck (A) and right upper limb (B) show hypertrophy of the right neck muscles and atrophy of the right brachioradialis, respectively. A cross-section of the chest CT image (C) and a 3D reconstruction of the CT image from the neck to the chest (D) are shown. Bilateral scapulae and clavicles were removed from the 3D reconstructed CT image. Fractures in bilateral first ribs and callus formation (arrows) are observed. The callus is dominant on the right side. Fractures and callus formation (arrowhead) are also found in the left second rib.

One month after admission, he abruptly noticed that he could not raise his right arm, and was referred to our neurology department. He exhibited intermittent involuntary movements such as irregular rotation, flexion, and extension of the neck. His neck muscles showed abnormal hypertonia and hypertrophy (Figure 1A). Atrophy of the right brachioradialis (Figure 1B) and carpi extensor muscles was observed. An examination revealed weakness of the right deltoid (Medical Research Council, or MRC, grade 3/5), brachioradialis (MRC grade 3/5), and wrist dorsiflexors (MRC grade 1/5). Reflexes were normal except for absent right triceps and brachioradialis reflex responses. The sensory examination was normal. Chest computed tomography showed fractures of the first rib on both sides, the second rib on the left side, and the left coracoid process, with callus formation (Figures 1C, 1D). There were no particular abnormalities in cervical spine MRI findings.

**Figure 2:**
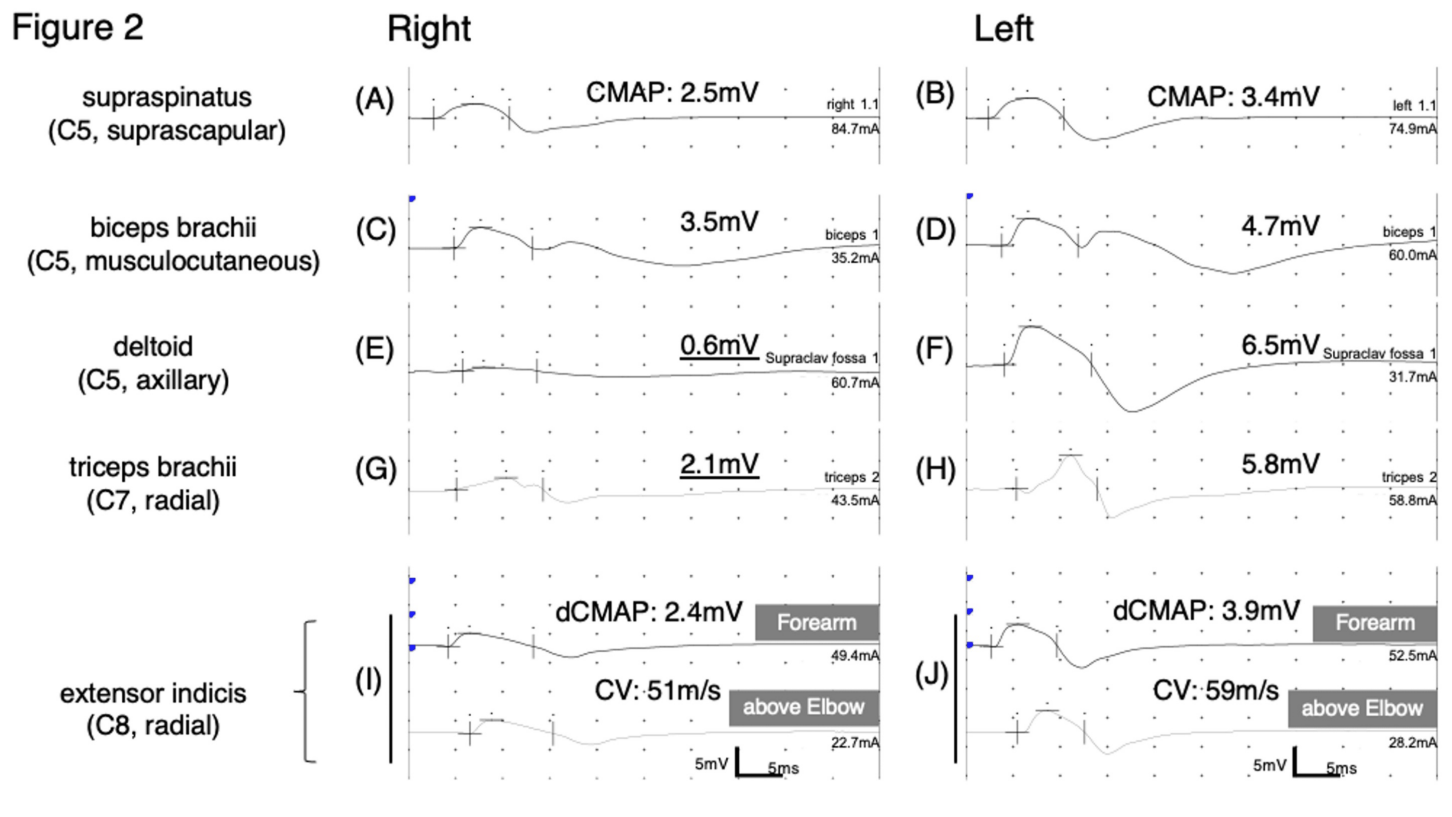
Nerve conduction studies Motor nerve conduction studies of bilateral supraspinatus (A, B), biceps brachii (C, D), deltoids (E, F), and triceps brachii (G, H) recorded by stimulating the Erb point are shown. A marked decrease in CMAP is observed in the right deltoid muscle, but the decreases in amplitude in the other C5 segment-innervated muscles, supraspinatus and biceps brachii, are not apparent. A decrease in CMAP is also observed in the right triceps brachii muscle, which is innervated by the radial nerve. Motor nerve conduction studies of bilateral radial nerves recorded in the extensor indicis (I, J) are shown. A slight decrease in CMAP is observed in the right extensor indicis. CMAP: compound muscle action potential; dCMAP: distal CMAP; CV: conduction velocity

**Figure 3:**
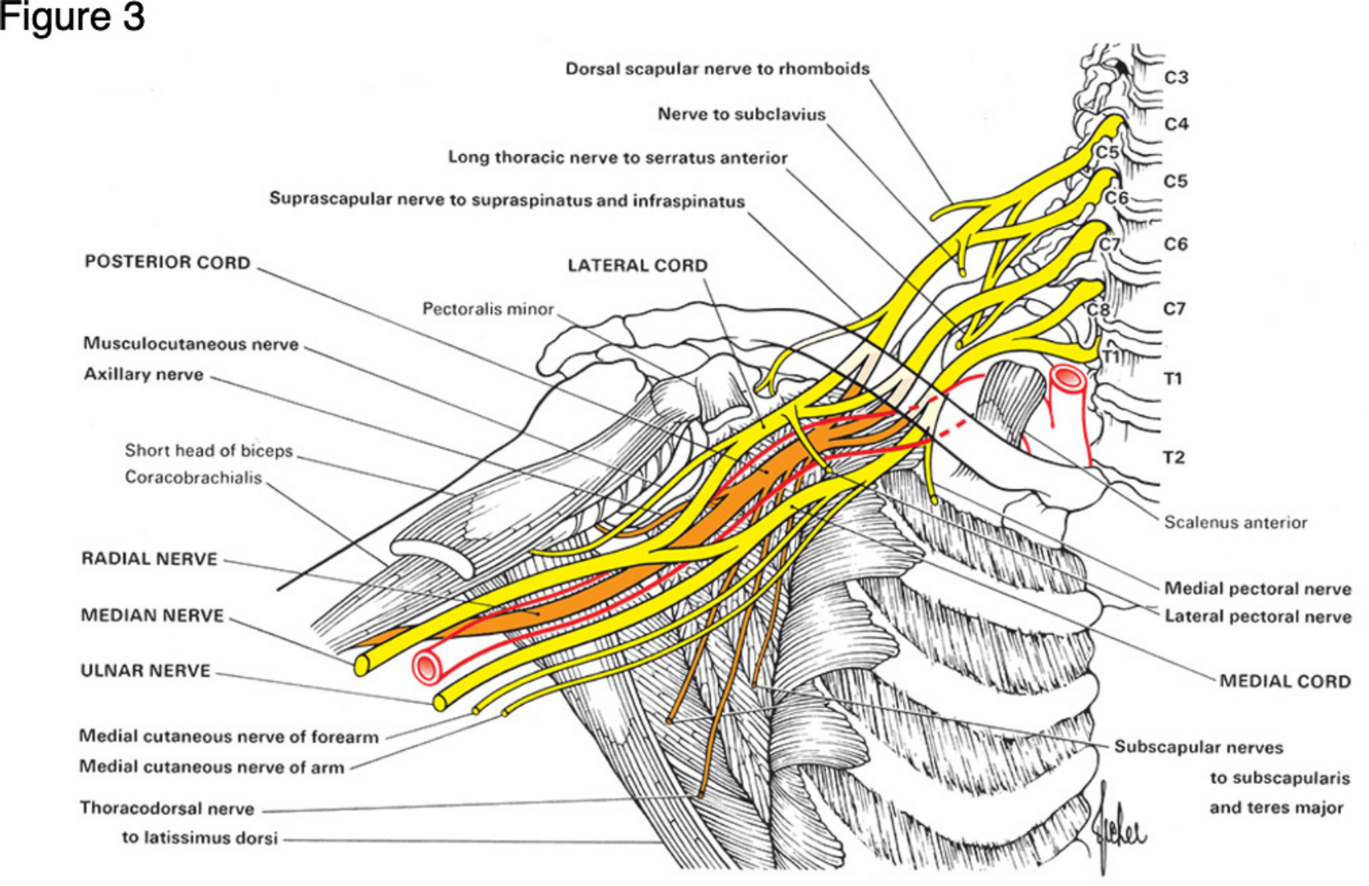
The brachial plexus In this case, impairment of muscles innervated by the radial nerve and the axillary nerve was observed, suggesting damage to the posterior cord of the brachial plexus. The posterior cord runs posterior to the second part of the axillary artery. Published in Aids of the Examination of the Peripheral Nervous System, Sixth Edition (O'Brien M, ed.), page 6, Elsevier (2023). Used with permission of Elsevier, all rights reserved.

The nerve conduction study (Figure 2) showed decreased compound muscle action potentials (CMAP) in the right deltoid and triceps brachii, but normal findings in the other muscles innervated by the right C5 segment. Motor nerve conduction studies of the right median and ulnar nerves revealed no abnormalities. Sensory nerve conduction studies were normal except for decreased sensory action potential of the right radial nerve measured at the snuff box. Based on the involvement of muscles innervated by the right axillary and radial nerves and presence of callus at the first rib, a diagnosis of right posterior cord entrapment was made (Figure 3) [4]. The patient was discharged home because he could not endure a long stay in the psychiatric ward. He was referred to a local orthopedic hospital for the brachial nerve injury.

## Discussion

Traumatic fractures of the first rib are rare because of the anatomical position and the short and thick bone. However, first-rib stress fractures sustained in various sports such as baseball, basketball, and weightlifting have been documented. Stress fractures have been reported to occur due to shear force applied to the subclavian groove as a result of contraction of the anterior and middle scalene muscles, the serratus anterior muscle, and the internal intercostal muscles attached to the first rib [5]. Stress fractures of the ribs often form nonunion calluses [1]. A previous study reported undiagnosed first-rib fractures in 0.1% of young healthy men [6], with the majority expected to be asymptomatic.

To our knowledge, an association between neck involuntary movements and stress fractures of the ribs has not been reported. In the present case, the intermittent strong contraction of muscles due to dyskinesia in the neck may have caused the stress fractures.

Among previous case reports of sports-related stress fractures of the first rib with brachial plexopathy, two reports described sensory disturbance on the ulnar side of the hand [1,7]. Another report of a case with a motor complication showed weakness of the muscles innervated by the radial nerve and surgical findings of compression of the middle trunk of the brachial plexus by a bone callus [2]. Brachial plexus entrapment is rare, and most cases involve damage to the lower brachial plexus, such as that seen in true neurogenic thoracic outlet syndrome (TN-TOS). TN-TOS is caused by the cervical rib or by a cord between the elongated transverse process of C7 and the first rib. The condition mainly shows T1 symptoms, such as thenar muscle atrophy, due to damage to the anterior rami of T1>C8 or lower trunk of the brachial plexus [8]. Considering the pattern of motor neuropathy in the present case, it is possible that brachial plexus injury, especially motor neuropathy, due to callus formation from the stress fracture of the first rib occurs more frequently in the posterior cord. This is suggested to be because the brachial plexus is entrapped by a swollen bone callus just distal to the scalene triangle, making the posterior cord located dorsal to the axillary artery more susceptible to injury.

## Conclusions

Here, we reported a case of posterior cord plexopathy caused by stress fractures of the first rib with callus formation, presumably as a result of tardive dyskinesia. Neurologists and physicians should recognize stress fracture of the first rib as a rare but an important cause of brachial plexopathy.
